# The impact of cognitive behavioral therapy for insomnia on cognitive performance and amyloid beta in older adults: A randomized controlled trial

**DOI:** 10.1002/alz.71591

**Published:** 2026-06-15

**Authors:** Catherine F. Siengsukon, Lauren K. Hand, Eryen Nelson, Allison Glaser, Rebecca Ludwig, Julia A. Russell, Milind A. Phadnis, Junqiang Dai, Jared Bruce, Eric D. Vidoni, Michelle Drerup, Jill Morris, Jeffrey M. Burns

**Affiliations:** ^1^ Department of Physical Therapy Rehabilitation Science, and Athletic Training University of Kansas Medical Center Kansas City Kansas USA; ^2^ Department of Medicine Division of General Internal Medicine University of Washington Seattle Washington USA; ^3^ Department of Psychology University of Kansas Lawrence Kansas USA; ^4^ Department of Biostatistics & Data Science University of Kansas Medical Center Kansas City Kansas USA; ^5^ Department of Biomedical and Health Informatics University of Missouri‐Kansas City School of Medicine Kansas City Missouri USA; ^6^ Departments of Neurology and Psychiatry University Health Kansas City Missouri USA; ^7^ University of Kansas Alzheimer's Disease Research Center Fairway Kansas USA; ^8^ Neurological Institute Sleep Disorders Center Cleveland Clinic Cleveland Ohio USA; ^9^ Department of Neurology University of Kansas Medical Center Kansas City Kansas USA

**Keywords:** Alzheimer's disease, beta‐amyloid, cognitive behavioral therapy, cognitive performance, dementia, insomnia

## Abstract

**INTRODUCTION:**

Insomnia is associated with increased risk for Alzheimer's disease (AD). It is unknown how cognitive behavioral therapy for insomnia (CBT‐I) impacts two hallmarks of AD progression, cognitive performance and beta‐amyloid (Aβ) burden.

**METHODS:**

Cognitively normal older adults with symptoms of insomnia were randomized into CBT‐I treatment (*n* = 100) or control (*n* = 100) groups. Cognitive performance was assessed at baseline, 6‐weeks, and 1‐year (1 year). Aβ burden was assessed in a subsample (*n* = 50).

**RESULTS:**

No differences were observed between groups in change in cognitive performance, including speed of information processing (mean difference, 0.017; 95% confidence interval [CI], −0.1036 to 0.1376; p = 0.78), executive function (−0.0881; 95% CI, −0.2945 to 0.1182; *p* = 0.40), and memory (0.4068; 95% CI, −2.3965 to 3.2101; *p* = 0.77). No group differences were observed in Aβ deposition.

**DISCUSSION:**

CBT‐I did not improve cognitive performance or Aβ deposition by one year. Longer follow up is needed to understand the potential impact of CBT‐I on AD risk.

**CLINICAL TRIAL REGISTRATION:**

The study was registered on clinicaltrials.gov (NCT03954210) on 5/17/2019.

## Highlights

- Insomnia has previously been associated with increased risk for dementia.
- Insomnia treatment did not improve cognitive performance or beta‐amyloid levels in older adults.
- Insomnia treatment improved insomnia and sleep self‐efficacy.
- More research is needed to understand if insomnia treatment impacts dementia risk.

## 1 BACKGROUND

The number of older Americans with Alzheimer's disease (AD) is growing, expecting to reach 13.8 million by 2060.^1^ Though some reports suggest a slowing of age‐specific prevalence,^1^ it is insufficient to meet the demands of a growing older adult population, thus more prevention efforts are needed. Older adults with insomnia have a 1.49 to 2.39‐fold higher risk for AD than those without insomnia,^2^, ^3^ and about 15% of AD is attributable to sleep problems.^4^ While the relationship between AD and sleep disturbances is bidirectional,^5^ the relationship between insomnia and cognitive decline prior to AD suggests insomnia as a target for AD prevention. For instance, insomnia moderates the impact of beta‐amyloid (Aβ) accumulation on cognitive decline prior to dementia, such that those with elevated Aβ and insomnia experience a more rapid cognitive decline than those with just elevated Aβ.^6^ Therefore, intervening to treat insomnia before cognitive decline presents an opportunity to disrupt the insomnia‐cognitive decline cycle and aid in AD prevention.

While the mechanisms through which AD and insomnia are linked are unclear, insomnia is related to two AD hallmarks: cognitive impairment and Aβ accumulation. Cross‐sectionally, middle‐aged and older adults that met criteria for insomnia disorder performed worse on declarative memory tasks than those with lesser or no insomnia symptoms.^7^ Moreover, a longitudinal study of middle‐aged and older adults found that those who worsened in insomnia symptoms were more likely to report subjective memory decline.^8^ One hypothesized mechanism for the link between AD and insomnia is via slow wave sleep (SWS) disruption. SWS is non–rapid eye movement (non‐REM) sleep that displays prominent roles in memory consolidation^9^ and Aβ clearance.^10^ Insomnia,^11^ cognitive decline,^12^ and Aβ elevation^13^ are all associated with declines in SWS. In fact, the impairment Aβ burden has on memory consolidation has shown to be mediated by disruptions in slow wave activity.^14^ Thus, improving SWS may have the potential to impact cognitive function.

Cognitive behavioral treatment for insomnia (CBT‐I) is an effective treatment for insomnia^15^, ^16^, ^17^ with better long‐term outcomes than pharmacotherapy.^18^, ^19^ CBT‐I is a multicomponent treatment that involves strategies to entrain the circadian rhythm and increase sleep drive, counter negative thoughts about sleep, manage physiologic tension and psychologic arousal, and promote sleep hygiene.^20^ In those with insomnia, CBT‐I has shown to improve several aspects of sleep, including increased sleep efficiency, total sleep time, and SWS.^21^

Given the positive impact of CBT‐I on sleep, a critical opportunity exists to employ CBT‐I in those with symptoms of insomnia to prevent early AD pathology, but limited data exist. Two studies have reported cognitive outcomes following CBT‐I in those with mild cognitive impairment (MCI), with one reporting improvements to executive function performance^22^ and the other reporting no changes.^23^ Additionally, two trials that included younger adults^24^, ^25^ had mixed results of CBT‐I on cognitive function with one randomized controlled trial (RCT) reporting improved cognitive complaints but no objective performance improvements^24^ and the other single‐arm trial reporting improved objective cognitive performance.^25^ One small trial (*n* = 31) in older adults (≥55 years) without dementia found no benefits to vigilance from CBT‐I compared to a pharmaceutical intervention, although no other cognitive domains were assessed.^26^ Furthermore, no reports have been made on the impact of CBT‐I on Aβ burden. Therefore, an appropriately powered RCT assessing the impact of CBT‐I on objective cognitive performance in healthy older adults is needed.

We conducted an RCT to evaluate the impact of CBT‐I on cognitive performance in older adults with insomnia symptoms. We hypothesized that CBT‐I would improve cognitive performance, specifically speed of information processing (primary outcome), executive function, and memory, compared to active control conditions. Additionally, we hypothesized that an increased time spent in SWS would be positively associated with improvements in cognitive performance, and that CBT‐I would slow the rate of Aβ deposition.

## 2 METHODS

**RESEARCH IN CONTEXT**

1. **Systematic review**: Given the existing data on the relationship between insomnia and dementia risk, the authors reviewed literature for trials evaluating the impact of cognitive behavioral therapy for insomnia (CBT‐I) on cognitive outcomes and beta‐amyloid accumulation. While a few trials have analyzed cognitive outcomes, very limited evidence exists on the impact of CBT‐I on cognition and beta‐amyloid accumulation in older adults without dementia.
2. **Interpretation**: Our data suggest CBT‐I does not impact cognitive performance or beta‐amyloid accumulation in cognitively normal older adults. However, CBT‐I improved self‐reported insomnia and depression symptoms. Given that insomnia is associated with increased risk of dementia, CBT‐I may benefit on long‐term dementia risk.
3. **Future directions**: Longer follow‐up is needed to understand if the benefits to insomnia and sleep self‐efficacy from CBT‐I influence dementia risk. Future investigation of the impact of CBT‐I on cognitive performance and beta‐amyloid accumulation within those with mild cognitive impairment is also warranted.

The Sleep Intervention to Enhance Sleep and reduce beta Amyloid (SIESTA) study was a randomized controlled trial employing CBT‐I in cognitively normal older adults with symptoms of insomnia, the protocol of which has been fully described previously.^27^ The aims of the trial were as follows: Aim 1, Evaluate the impact of CBT‐I on cognitive outcomes; Aim 2, Assess the associations between changes in sleep and changes in cognitive function; Exploratory Aim, Examine the impact of CBT‐I on Aβ deposition. All study assessments took place at the University of Kansas Medical Center (KUMC) from August 2019 to April 2025. The study was registered on clinicaltrials.gov (NCT03954210) and approved by the KUMC Human Subjects Committee (HSC# STUDY00143545).

### 2.1 Participants

Individuals were eligible to participate if between 60 and 85 years of age, cognitively normal (Mini‐Mental State Examination ≥ 25^28^, ^29^ and AD8 Dementia Screening < 2^30^), reporting chronic symptoms of insomnia (i.e., difficulty falling asleep, maintaining sleep, or waking up too early at least three times a week for 3 consecutive months), and scoring 10 or greater on the Insomnia Severity Index (ISI).^31^ Exclusion criteria included having another known untreated sleep disorder (i.e., obstructive sleep apnea [OSA], periodic limb movements in sleep [PLMS]) or signs of another untreated sleep disorder (e.g., sleep apnea or Apnea–Hypopnea Index ≥ 15, restless legs syndrome or periodic limb movements with arousals ≥ 5/hour); taking benzodiazepines, non‐benzodiazepines, melatonin supplements, or agonists for insomnia, indications of severe depression or suicidal ideation per the Patient Health Questionnaire‐9 (PHQ‐9)^32^; history of drug or alcohol abuse, history of nervous system disorder, severe mental illness, history of learning disability or attention‐deficit/hyperactivity disorder, current or history of shift work, currently receiving CBT‐I, and sight or hearing difficulty.

Participants were recruited from the University of Kansas Alzheimer's Disease Research Center (KU ADRC), Alzheimer's and research registries, radio/print advertisements, community outreach activities, and social media.^33^ Rather than confirmatory hypothesis testing, the primary emphasis of this pilot study was effect size estimation based on 95% confidence intervals to inform future studies. We had the capacity to enroll *N* = 200 subjects for this pilot study, and we calculated that with this sample size we had 80% power to detect a standardized effect size of 0.31 between the CBT‐I and AC groups with a two‐sided two‐sample Z‐test of two means using a type‐I error of 5%. Since previous studies in this area of research had variable sample sizes and many were small sample studies, we were satisfied with our ability to enroll 200 subjects so that hypothesized mean differences between the two groups could be detected even after adjusting for covariates. The exploratory aim subsample goal was *n* = 50 to allow for effect size estimation within 0.65 SD. All participants underwent informed consent. Details on recruitment were previously published.^33^

### 2.2 Randomization

Participants were randomized 1:1 into either the CBT‐I group or active control (AC) group. The statistician generated and kept the randomization sequence, which was conducted using a random/pseudo‐random number generator and random block sizes of 4, 6, 8, and 10.^27^ The CBT‐I group underwent 6 weeks of standardized CBT‐I^20^ through weekly one‐on‐one sessions with masters‐ or doctoral‐level trained personnel. Sessions typically lasted 45‐60 minutes each. The intervention has been previously described,^27^ and in brief, the sessions focused on the following topics: (1) Time in bed restriction, sleep hygiene, stimulus control; (2) Relaxation and arousal reduction through breathing techniques; (3) Mindfulness and addressing any negative sleep beliefs with cognitive therapy; (4) Arousal reduction through progressive muscle relaxation; (5) Review and reinforcement of needed techniques; and (6) Maintenance and relapse prevention. The focus of Session 6 was to normalize occasional challenges sleeping and potential relapse of insomnia, emphasize their knowledge and skills to manage their sleep independently, and collaboratively completing a document on strategies that were most effective for them to use as a resource as needed. While the sessions began in person, they were moved to virtual video conferencing in March 2020 due to coronavirus disease 2019 (COVID‐19) pandemic, though the eligibility criteria and content delivered remained the same. Telehealth‐delivered CBT‐I has shown to produce similar outcomes as traditional in‐person CBT‐I regarding sleep‐related outcomes.^34^ After the 6‐week program was complete, the CBT‐I group received monthly phone calls with research personnel trained in motivational interviewing and behavior change strategies to discuss and aid in maintenance of strategies until one year following the initial visit. However, improvements in sleep outcomes remain up to 10 years following CBT‐I without “booster” sessions.^35^ Alternatively, the AC group initially consisted of 6 weekly 45‐ to 60‐minute sessions that included stretching and thinking activities (i.e., puzzles, board games, or Nintendo Wii) one‐on‐one with study personnel to control for contact and socialization. However, with needing to implement the intervention via video conferencing in March 2020 due to the pandemic, engaging with the participants to the same degree using video games was challenging. Thus, we switched the AC to stretching and lifestyle education to more closely mimic the interaction with study personnel experienced by the CBT‐I group. Each AC session started and ended with stretching, and lifestyle education was provided (i.e., general information about sleep, environmental factors affecting sleep, how alcohol, nicotine, and screen time can affect sleep, diet, exercise, and mattresses and pillows). The Principal Investigator (PI) randomly selected 25% of participants in the CBT‐I group to review session recordings and completed a fidelity checklist. Additionally, the interventionists performed a self‐check of content provided for each session following completion of the session using a standardized form. Adverse events reported from participants were recorded and subsequently graded as related/not related to the study testing/intervention and as anticipated/unanticipated by the PI, and a summary of all adverse events was reported biannually to the safety officer and National Institutes of Health (NIH) Program Administrator.

### 2.3 Data collection

Study visits occurred at baseline, 6 weeks from baseline (i.e., post‐intervention), and 1 year from the end of the intervention. While blinding interventionists and participants was not possible due to the nature of a behavioral intervention, the study personnel conducting screening, enrolling, and data collection at each study visit were blinded to group allocation. The following procedures were performed at each visit:

*Demographic and health history* information was gathered via self‐report including smoking status, age, education, race/ethnicity, marital status, sex/gender, and employment status.

*Cognitive performance* was assessed using a battery of cognitive tests tapping speed of information processing, memory, and executive function. Speed of information processing was measured using the Continuous Performance Test (CPT‐3)^36^ and coding test from Continuous Performance Test (CPT‐3).^37^ Memory was evaluated using the RBANS.^37^ Executive function was assessed using the Stroop Test and Neuropsychological Assessment Battery (NAB) Digits Forward and Backward.^38^ During the COVID‐19 shutdown, cognitive performance testing was temporarily moved virtually, which has shown to produce comparable results as in‐person testing.^39^

*Sleep measures* were evaluated using one overnight polysomnography (PSG). Standardized protocols were used to prepare the participants^40^ and place six electroencephalogram sensors to detect brain waive activity.^41^ Total sleep time (TST); time in bed (TIB); sleep efficiency (SE); wake after sleep onset (WASO); sleep onset latency (SOL); sleep stages 1 (N1), 2 (N2), and 3 (N3); and rapid eye movement sleep (REM) were determined using standardized scoring procedures.^40^

*Additional psychosocial measures* were collected due to their known associations with cognitive function including depression using the PHQ‐9,^32^ anxiety using the Generalized Anxiety Disorder Assessment (GAD‐7),^42^ and Sleep Self‐Efficacy Scale (SSE).^43^

### 2.4 Exploratory data collection

*Apolipoprotein ε (APOE) genotyping* was completed using restriction enzyme isotoping on blood samples collected in Acid Citrate Dextrose Vacutainer tubes and processed for DNA extraction. APOE was characterized as a covariate based on the presence or absence of APOE4.

*Magnetic resonance imaging (MRI) and positron emission tomography (PET) Imaging* was completed to evaluate Aβ deposition. The methods have been previously detailed.^44^ In short, ^18^F‐AV‐45 (Florbetapir) PET images were obtained using a GE 64 slice PET/computed tomography (CT) MIDR scanner. Florbetapir (370 MBq) was administered once to participants via IV catheter, and two 5‐minute PET frames were acquired continuously about 45 minutes following the injection. The average PET signal summed, attenuation corrected, and smoothed using a 6 mm full‐width, half‐maximum kernel. MRI was performed within two weeks of the PET imaging for a high resolution T1‐weighted for detailed anatomy (Siemens 3.0T Skyra, MP‐RAGE; 1 × 1 × 1 mm voxels; repetition time [TR]  =  2300 ms, echo time [TE]  =  2.98 ms, inversion time [TI]  =  900, field of view [FOV]  =  240 × 256, 1 mm slice thickness, flip angle 9°). We used SPM25{(https://www.fil.ion.ucl.ac.uk/spm) and CAT12 (neuro‐jena.github.io) for imaging analyses.^45^, ^46^ The anatomical image was first coregistered to the PET image. Next, the imaging was segmented into gray matter, white matter, and cerebrospinal fluid in native imaging space. An accompanying Neuromorphometrics regional parcellation atlas was generated. To explore global Aβ burden we implemented an approach adopted and modified from the Global Alzheimer's Association Interactive Network (GAAIN) Centiloid Project.^47^ The whole cerebellum served as the reference region. Each PET image was divided by the mean reference signal to generate a standardized uptake value ratio (SUVR) image. The SUVR image was then converted to Centiloids (CL).^47^, ^48^ To ensure cortical specificity, we multiplied the gray matter segmentation with the inverse‐warped GAAIN CL region of interest (ROI), yielding a participant‐specific cortical ROI representing brain regions with high Aβ burden in AD. The average CL value within the ROI was calculated for each person and analyzed outside of imaging space.

### 2.5 Statistical analysis

Participant characteristics were described using means and standard deviation or count and frequencies. Descriptive characteristics were compared between groups using independent sample t‐tests and chi‐squared or Fisher exact tests (based on cell counts) for continuous and categorial variables, respectively. Speed of Information Processing score was calculated as the mean of two standardized component scores: the Hit Reaction Time (HRT) Z‐score and the Coding Total Z‐score. Executive Function score was calculated as the mean of three standardized component scores: the Stroop Interference Z‐score, Digits Forward Z‐score, and Digits Backward Z‐score. All the standardized Z‐scores were computed using the baseline mean and standard deviation of the raw scores. All component test scores were standardized (Z‐scores) using the baseline mean and standard deviation of the raw scores. For measures where higher raw scores indicated poorer performance (i.e., HRT and Stroop Interference), Z‐scores were multiplied by −1 after standardization so that higher values consistently reflected better cognitive performance. Thus, higher composite scores indicate better cognitive performance. The delayed memory score from RBANS was used as the primary memory outcome. Linear mixed‐effects models were fitted to examine the efficacy of CBT‐I on cognitive performance with (i.e., APOE4 genotype, age, sex, and education level) or without adjusting for covariates. Analyses were run initially as intention‐to‐treat using the linear mixed‐effects models, which handles missing data assuming the missing is at random. Statistical assumptions related to linear mixed models were assessed using diagnostic plots such as residual vs fitted values plot, normal Q‐Q plot and by means of a random effects diagnostic plots for assessing normality of estimated random effects, and all assumptions were found to be appropriate. The analyses were repeated using only those with complete data for a given outcome (i.e., completers analysis) and then comparing responders versus non‐responders based on the change of ISI (responder = a decrease from baseline of six or more^49^ or total score < 10) where a comparison of the changes between groups were conducted using two‐sample t‐test (where data was normally distributed) and Wilcoxon rank sum test (where data was not normally distributed). Additionally, a post‐hoc subgroup analysis and interaction analysis was performed to assess the efficacy of CBT‐I on cognitive performance in those with or without APOE4 genotype. To explore the association between sleep changes and cognitive function, linear mixed‐effects models were implemented. Between group differences of Aβ deposition were tested using two sample *t*‐tests; linear regression models were fitted to adjust for baseline amyloid burden and SWS. A comparison of the changes in survey data outcomes (i.e., ISI, GAD7, PHQ‐9, and SES) and sleep characteristics between the CBT‐I and the AC group were conducted using two‐sample *t*‐test and Wilcoxon rank sum test. Significance was set at *p* < 0.05. Statistical analyses were conducted using SAS Institute Inc. SAS 9.4. Cary, NC: SAS Institute Inc.; 2013.

## 3 RESULTS

**FIGURE 1:**
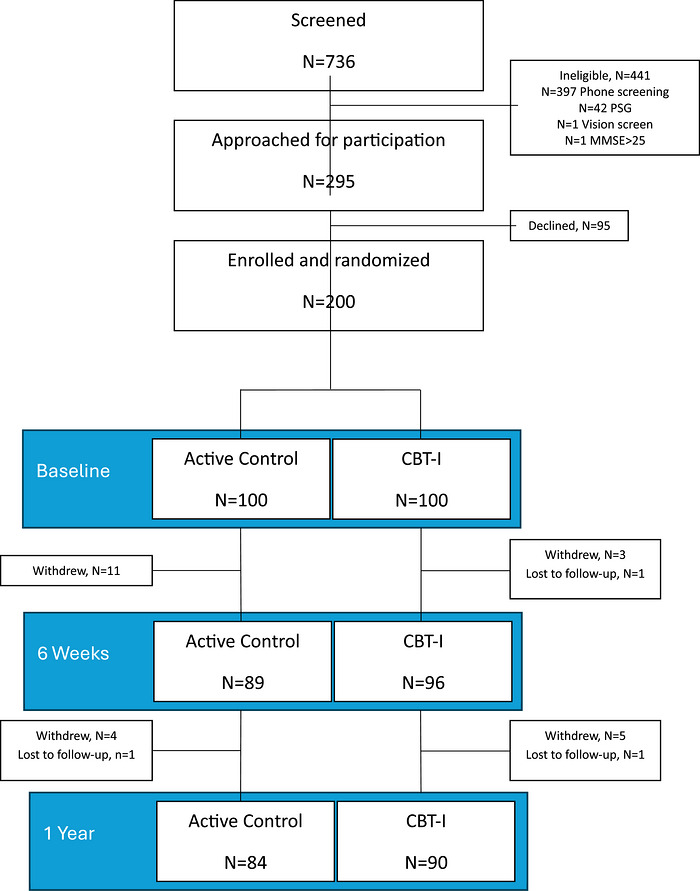
Consort diagram. CBT‐I, cognitive behavioral therapy for insomnia; MMSE, Mini‐Mental State Examination; PSG, polysomnography.

Two hundred participants were enrolled and randomized (*n* = 100 for both groups, Figure 1). Participants were 69.4 ± 5.5 years of age, 79% female, 95% non‐Hispanic/Latino, and 87% White. There were no differences between groups for baseline characteristics (Table 1). Fifteen participants did not complete the 6‐week visit (*n* = 11 for AC, *n* = 4 for CBT‐I) and 26 did not complete the 1‐year visit (*n* = 16 for AC, *n* = 10 for CBT‐I). Among those that attended the 6‐week study visit, intervention session attendance was high (95.0% for CBT‐I and 91.9% for AC). Eighteen participants (*n* = 9 for CBT‐I, *n* = 9 for AC) completed the intervention in‐person. Due to the COVID‐19 shut down, five participants (*n* = 3 for CBT‐I, *n* = 2 for AC) that had begun the intervention in‐person, completed the intervention virtually as did the remainder of the participants. Six adverse events occurred related to the data collection for the study (two in CBT‐I, one in AC, and three during screening in participants who were not randomized) of which two were expected (i.e., bruising at PET injection site, disturbed sleep/discomfort/anxiety from PSG) and four were unexpected, including an allergic reaction to the gel used to secure the PSG sensors, hair and skin damage from the PSG adhesives, and experiencing aura from the computer screen when completing CPT‐3. At 6 weeks, 84.4% (*n* = 81) of CBT‐I and 42.7% (*n* = 38) of AC were categorized as responders (*p* < 0.0001). At 1 year, 81.1% (*n* = 73) of CBT‐I and 61.9% (*n* = 52) of AC were categorized as responders (*p* = 0.005). Neither group experienced any changes in PSG‐measured sleep characteristics at 6 weeks or 1 year (Supplemental Table S1). Three participants in the AC group reported they had discussed sleep‐related issues with a physician at the post‐intervention reassessment, including two who discussed use of continuous positive airway pressure (CPAP). At the 1‐year reassessment, five participants in the AC group and four in the CBT‐1 group reported discussing sleep issues with a physician and/or therapist, including one participant in the AC group who reported a new diagnosis of sleep apnea with CPAP prescription that had not yet begun. One participant from AC reported reading about and applying CBT‐I practices on their own. Eight participants in the AC group and three participants in the CBT‐I group reported taking a new medication or supplement for sleep between 6 weeks and 1 year.

**TABLE 1:** Participant characteristics

Characteristic	Active control group *N* (%) or mean ± SD	CBT‐I group *N* (%) or mean ± SD
*N*	100	100
Age (years)	69.1 ± 5.8	69.6 ± 5.3
**Sex**		
Female	81 (81%)	77 (77%)
Male	19 (19%)	23 (23%)
**Race**		
White	87 (87%)	87 (87%)
Black/African American	12 (12%)	11 (11%)
Asian	1 (1%)	1 (1%)
More than one race	0 (0%)	1 (1%)
**Ethnicity**		
Non‐Hispanic/Latino	94 (94%)	96 (96%)
Unknown	6 (4.7%)	4 (4%)
**Education**		
Less than high school	1 (1%)	1 (1%)
High school	0 (0%)	2 (2%)
Some college, no degree	9 (9%)	10 (10%)
Associate's degree/some college	6 (6%)	9 (9%)
Bachelor's degree	35 (35%)	30 (30%)
Master's degree	36 (36%)	40 (40%)
Doctoral level degree	13 (13%)	8 (8%)
**Smoking history**		
Current smoker	0 (0%)	0 (0%)
History of smoking	30 (30%)	36 (36%)
**APOE Genotype**		
ε4+	27 (27%)	35 (35%)
ε4‐	58 (58%)	57 (57%)
Missing	15 (15%)	8 (8%)
ISI	14.8 ± 3.6	14.9 ± 3.2
PHQ‐9	5.3 ± 3.1	5.2 ± 2.8
GAD‐7	2.9 ± 3.7	3.3 ± 3.3
SSE	18.7 ± 6.8	18.8 ± 6.5

*Notes* Between‐group differences in descriptive characteristics were detected using independent sample t‐tests and chi‐squared or fisher exact tests (based on cell counts) for continuous and categorial variables, respectively. No between‐group differences were detected.

Abbreviations: ISI, Insomnia Severity Index; GAD‐7, Generalized Anxiety Disorder Assessment; PHQ‐9, Patient Health Questionnaire; SSE, Sleep Self‐Efficacy Scale.

### 3.1 Aim 1: Efficacy of CBT‐I on cognitive performance

Unadjusted scores for the cognitive performance assessments are presented in Table 2. For the intention‐to‐treat analysis, unadjusted linear mixed‐effect models were fitted, and no statistically significant group differences were found between the CBT‐I and AC arms for any cognitive domain at post‐intervention or 1‐year follow‐up. For the primary outcome, speed of information processing, the estimated mean difference was 0.017 (95% CI, −0.1036 to 0.1376; p = 0.78), indicating no significant improvement attributable to the CBT‐I intervention. Similarly, results for executive function (mean difference, −0.0881; 95% CI, −0.2945 to 0.1182; *p* = 0.40) and memory domain (mean difference, 0.4068; 95% CI, −2.3965 to 3.2101; *p* = 0.77) were not statistically significant (Table 3).

**TABLE 2:** Cognitive performance assessments.

	Active control						CBT‐I					
Outcome	Baseline (N)	Baseline (mean ± SD)	6 Weeks (N)	6 Weeks (mean ± SD)	1 Year (N)	1 Year (mean ± SD)	Baseline (N)	Baseline (mean ± SD)	6 Weeks (N))	6 Weeks (mean ± SD)	1 Year (N)	1 Year (Mean ± SD)
**Speed of Information Processing Composite** ^†^	99	0 ± 0.8	84	0 ± 0.8	78	0.1 ± 0.8	98	0 ± 0.8	90	0 ± 0.8	84	0 ± 0.8
Hit Reaction Time (HRT)	99	55.4 ± 6.7	84	56.2 ± 6.9	81	55.3 ± 7.1	98	55.6 ± 8.3	90	55.8 ± 8.4	86	55.1 ± 8.2
HRT Standard Deviation (SD)	99	46.1 ± 6.5	84	44.2 ± 6.1	81	43.5 ± 5.1	98	46.1 ± 5.9	90	44 ± 6.3	86	43.4 ± 5
**Delayed Memory Index Score**	100	106.8 ± 10.9	89	109.1 ± 13	83	109.5 ± 13.1	100	104.5 ± 10.6	95	107.8 ± 11.5	89	106.4 ± 11.5
Immediate Memory Index Score	100	109 ± 14.9	89	109.9 ± 13.1	83	109.3 ± 15.3	100	106.1 ± 13.2	95	106.5 ± 13.8	89	103.9 ± 13.3
RBANS Total Scale	100	108.7 ± 13.2	89	111.2 ± 13.6	83	111.4 ± 14.2	100	105.9 ± 12.2	95	108.7 ± 12	89	107.6 ± 12.6
**Executive Function Composite** ^†^	98	0 ± 0.7	87	0.3 ± 1.1	82	0.2 ± 1	98	0 ± 0.7	93	0.1 ± 0.9	87	0.1 ± 0.8
Raw Color Score	98	69.7 ± 13.7	87	70.2 ± 12.9	82	69.9 ± 13.6	98	66.1 ± 9.8	93	68 ± 11.6	87	67.1 ± 10.9
Raw Interference Score	98	−1.6 ± 6.8	87	1.1 ± 12	82	0.8 ± 11	98	−1.6 ± 7.3	93	−0.7 ± 10.1	87	−1.1 ± 8.9
Interference T Score	98	48.2 ± 6.5	87	49.6 ± 7	82	49.3 ± 6.7	98	48 ± 5.9	93	48.1 ± 6	87	48.1 ± 6.2
Digits Forward Longest Span	100	6.6 ± 1.4	89	6.9 ± 1.4	84	6.7 ± 1.4	100	6.5 ± 1.3	95	6.8 ± 1.3	89	6.7 ± 1.2
Digits Backward Longest Span	100	5 ± 1.4	89	5 ± 1.4	84	5 ± 1.4	100	4.9 ± 1.2	95	5.1 ± 1.3	89	4.8 ± 1.3

*Note*: Unadjusted means ± SD are presented for each assessment.

Bolded outcomes are the primary outcomes assessed for memory, speed of information processing, and executive function.

Abbreviation: RBANS, Repeatable Battery for the Assessment of Neuropsychological Status.

**^†^** Composite scores were calculated by finding the mean of standardized component scores of related tests (i.e., the Hit Reaction Time (HRT) Z‐score and the Coding Total Z‐score for speed of information processing and the Interference Z‐score, Digits Forward Z‐score, and Digits Backward Z‐score for executive function).

**TABLE 3:** Intent‐to‐treat analysis for cognitive performance

Assessment	Outcome	Domain	Estimate	95% CI	*p*‐value
**Continuous Performance Test Hit Reaction Time (HRT) and RBANS Coding Total Score composite**	Speed Information Processing Composite	Speed of Information Processing, primary outcome	0.017	(−0.1036, 0.1376)	0.7811
Continuous Performance Test	Hit Reaction Time	Speed of Information Processing	−0.0921	(−1.2533, 1.0691)	0.8758
Continuous Performance Test	HRT Standard Deviation	Speed of Information Processing	−0.4153	(−1.6962, 0.8655)	0.523
**RBANS**	Delayed Memory Index Score	Memory	0.4068	(−2.3965, 3.2101)	0.7749
RBANS	Immediate Memory Index Score	Memory	−1.1263	(−4.5263, 2.2737)	0.5142
RBANS	RBANS Total Scale	Global Cognitive Functioning	−0.028	(−2.4699, 2.4139)	0.982
**Interference (Stroop) and Digits forward, Digits Backward composite**	Executive Function Composite	Executive Function	−0.0881	(−0.2945, 0.1182)	0.4005
Stroop Test	Raw Color Score	Executive Function	1.0664	(−0.7523, 2.8852)	0.2488
Stroop Test	Raw Interference Score	Executive Function	−1.4911	(−4.195, 1.2129)	0.278
Stroop Test	Interference T Score	Executive Function	−1.1191	(−2.5713, 0.333)	0.1301
NAB Digits Forward, Digits Backward	Digits Forward Longest Span	Executive Function	0.0381	(−0.2592, 0.3354)	0.8007
NAB Digits Forward, Digits Backward	Digits Backward Longest Span	Executive Function	0.0701	(−0.243, 0.3833)	0.659

*Note*: An unadjusted linear mixed‐effect model was run to detect differences between groups in change in cognitive performance. Bolded outcomes are the primary outcomes assessed for memory, speed of information processing, and executive function.

Abbreviations: CI confidence interval; NAB, Neuropsychological Assessment Battery; RBANS‐Repeatable Battery for the Assessment of Neuropsychological Status.

Linear mixed‐effect models adjusting for APOE4 genotype, age, sex, and education level also yielded no significant treatment effects. Similarly, no differences were detected when comparing CBT‐I and AC changes only in the completers or assessing differences between responders and non‐responders. Additionally, no differences were detected in the post‐hoc subgroup analysis looking only at those with or without APOE4 genotype and the group*APOE4 interaction term was not statistically significant.

### 3.2 Aim 2: Association between sleep changes and cognitive function

Table 4 shows the results from linear mixed‐effect modeling of sleep stage changes. There were no significant associations between change in SWS and cognitive outcomes. A statistically significant association was observed between change in REM sleep and executive function, where an increase in REM sleep was associated with a modest reduction in executive functioning (estimate, −0.003; 95% CI, −0.005 to 0.000; *p*P = 0.024). Increases in REM sleep also showed associations with improvements in Raw Interference Score (estimate, −0.030; 95% CI, −0.058 to −0.002; *p* = 0.038) and declined Digits Backward Raw Score (estimate, −0.008; 95% CI, −0.015 to −0.001; *p* = 0.024). N2 sleep change showed a significant positive association with hit reaction time standard deviation, such that longer N2 duration is related to a higher variability of hit reaction time (estimate, 0.009; 95% CI, 0 to 0.017; *p* = 0.041). No other sleep‐cognition associations reached statistical significance.

**TABLE 4:** Association between change in sleep measures and change in cognitive function

Outcome	N1		N2		N3/SWS		REM	
	Estimate (95% CI)	*p*‐value	Estimate (95% CI)	*p*‐value	Estimate (95% CI)	*p*‐value	Estimate (95% CI)	*p*‐value
Hit Reaction Time (HRT)	−0.009 (−0.03, 0.013)	0.425	0.005 (−0.004, 0.013)	0.287	−0.006 (−0.019, 0.007)	0.341	−0.009 (−0.025, 0.008)	0.286
HRT Standard Deviation	−0.007 (−0.027, 0.013)	0.499	0.009 (0, 0.017)	0.041^*^	0 (−0.012, 0.013)	0.975	−0.006 (−0.022, 0.01)	0.448
Immediate Memory Index Score	0.03 (−0.03, 0.091)	0.327	0.014 (−0.011, 0.038)	0.27	−0.017 (−0.055, 0.02)	0.361	−0.013 (−0.06, 0.035)	0.591
Coding Total Score	0.001 (−0.021, 0.023)	0.903	−0.003 (−0.012, 0.006)	0.507	0.004 (−0.009, 0.018)	0.535	0.011 (−0.007, 0.028)	0.228
Delayed Memory Index Score	−0.038 (−0.086, 0.011)	0.127	0.007 (−0.013, 0.026)	0.486	−0.003 (−0.033, 0.027)	0.829	−0.025 (−0.063, 0.013)	0.202
RBANS Total Scale	0.004 (−0.041, 0.049)	0.861	0.004 (−0.014, 0.022)	0.632	−0.014 (−0.042, 0.014)	0.318	−0.011 (−0.047, 0.024)	0.529
Raw Color Score	0.012 (−0.019, 0.043)	0.459	−0.009 (−0.021, 0.004)	0.163	0.006 (−0.013, 0.025)	0.549	0.003 (−0.021, 0.027)	0.799
Raw Interference Score	0 (−0.035, 0.036)	0.991	0.002 (−0.012, 0.017)	0.763	−0.008 (−0.03, 0.015)	0.503	−0.03 (−0.058, ‐0.002)	0.038^*^
Interference T Score	0.001 (−0.026, 0.027)	0.969	0.005 (−0.006, 0.015)	0.377	−0.007 (−0.023, 0.009)	0.402	−0.013 (−0.033, 0.008)	0.22
Digits Forward Raw score	0 (−0.009, 0.009)	0.971	0 (−0.003, 0.004)	0.91	−0.002 (−0.008, 0.003)	0.418	−0.001 (−0.008, 0.006)	0.693
Digits Backward Raw score	−0.003 (−0.012, 0.006)	0.483	−0.002 (−0.006, 0.001)	0.234	−0.004 (−0.009, 0.002)	0.17	−0.008 (−0.015, ‐0.001)	0.024^*^
Speed Information Processing	−0.001 (−0.002, 0.001)	0.316	0 (−0.001, 0)	0.151	0 (0, 0.001)	0.328	0 (−0.001, 0.002)	0.641
Executive Function	0 (−0.003, 0.003)	0.984	0 (−0.001, 0.001)	0.927	−0.001 (−0.003, 0.001)	0.206	−0.003 (−0.005, 0)	0.024^*^

* *Note*: Linear mixed‐effect models were run to evaluate the associations between change in sleep measures and change in cognitive function. Sleep stage variables were characterized in minutes.

Abbreviation: RBANS, Repeatable Battery for the Assessment of Neuropsychological Status.

*p < 0.05.

### 3.3 Exploratory aim: Effect of CBT‐I on Aβ deposition

Aβ was analyzed in 43 participants at baseline (*n* = 21 for AC, *n* = 22 for CBT‐I). The entire cohort had an Aβ of 50.1 ± 37.3 CL at baseline, with 62.8% having > 30 CL, indicative of Aβ pathology,^50^ with no difference between groups (*p* > 0.05). There was no significant difference in Aβ accumulation between groups at 1‐year follow‐up (Table 5). The mean change in Aβ burden was 1.4 CL (95% CI, ‐2.9 to 5.7) in AC and 0.1 CL (95% CI, ‐4.7, 5.0) in CBT‐I. The between‐group mean difference in change score was not significant (−1.25; 95% CI, −7.47 to 4.98; *p* = 0.76). When adjusting for baseline amyloid burden and SWS, the linear model estimate remained non‐significant (estimate, −0.98; 95% CI, −7.35 to 5.39; *p* = 0.76).

**TABLE 5:** Change in beta amyloid between groups

	Active control (1 year baseline)		CBT‐I (1 year baseline)		CBT‐I active control		CBT‐I active control (linear model adjusting for baseline SWS and baseline amyloid burden)	
Outcome	Mean change (95% CI)	Paired t‐test *p*‐value	Mean change (95% CI)	Paired t‐test *p*‐value	Mean difference (95% CI)	Two sample t‐test *p*‐value	Model estimate (95% CI)	Linear model *p*‐value
Centiloid Score	1.39406 (−2.91048, 5.6986)	0.5049	0.14693 (−4.6851, 4.97896)	0.9496	−1.24713 (−7.47104, 4.97678)	0.6866	−0.98028 (−7.35421, 5.39364)	0.7563

Abbreviations: CI, confidence interal; CBT‐I, Cognitive Behavioral Therapy for Insomnia; SUVR, standardized uptake value ratio; SWS, slow wave sleep.

### 3.4 Analysis of additional psychosocial measures

The CBT‐I group showed a greater reduction in ISI scores (mean change, −8.2 ± 4.4; 95% CI, −9.23 to −7.26) compared to the AC (−5.6 ± 4.9; 95% CI, −6.64 to −4.5; *p* = 0.0003; Table 6). Similarly, the CBT‐I group had larger improvements in the SSE score (6.7 ± 6.4 vs. 3.9 ± 6.9; *p* = 0.0067). Changes in PHQ‐9 and GAD7 were not different between groups.

**TABLE 6:** Between‐group difference in change in insomnia severity index and additional psychosocial measures (1 year minus baseline)

	Active control			CBT‐I				
Outcome	*N*	Mean ± SD	95% CI	*N*	Mean ± SD	95% CI	*p*‐value	Test
Insomnia Severity Index (ISI)	84	−5.571 ± 4.939	(−6.643, −4.5)	90	−8.244 ± 4.708	(−9.23, −7.258)	0.0003^*^	T‐test
Patient Health Questionnaire (PHQ)	84	−1.917 ± 2.725	(−2.508, −1.325)	90	−2.511 ± 3.177	(−3.177, −1.846)	0.0992	Rank sum
Generalized Anxiety Disorder (GAD7)	83	0.253 ± 2.862	(−0.372, 0.878)	90	−0.5 ± 2.896	(−1.107, 0.107)	0.1585	Rank sum
Sleep Self‐Efficacy Scale (SSE)	84	3.94 ± 6.869	(2.45, 5.431)	90	6.711 ± 6.446	(5.361, 8.061)	0.0067^*^	T‐test

Abbreviations: CI, confidence interal; CBT‐I, Cognitive Behavioral Therapy for Insomnia; SD, standard deviation.

* *p* < 0.05.

## 4 DISCUSSION

We conducted a randomized controlled trial investigating the impact of CBT‐I on cognitive performance and Aβ deposition in cognitively normal older adults with insomnia symptoms. We found no differences between the CBT‐I and AC groups regarding cognitive performance or Aβ deposition up to one year following the intervention. Furthermore, contrary to our hypothesis, change in SWS was not related to change in Aβ deposition; however, small associations were observed between change in N2 and REM with change in several cognitive performance scores. The CBT‐I group had a better improvement in ISI and SSE compared to the AC group after one year. Overall, while CBT‐I did not result in changes to cognitive performance or Aβ deposition, it was effective at improving insomnia and sleep self‐efficacy.

Previous reports on the impact of CBT‐I on objective cognitive function have been mixed with some reporting benefits^22^, ^25^, ^51^ while others reported null results.^24^, ^26^, ^52^ Important differences exist between those reporting positive findings and our study. First, the sample sizes in the earlier trials with positive results were small (i.e., *n* = 10,^25^ 28,^22^ and 31^51^), one of which was a single‐arm pilot trial,^25^ thus the results needed to be corroborated with larger RCTs. By contrast, while two of the trials with null findings were also small (*n* = 31^26^ and 62^52^), ours and another with *n* = 410^24^ were adequately powered. Additionally, most prior studies included younger adults, with only three focused on older adults.^22^, ^23^, ^26^ The older adult cohort of those without dementia saw no benefit to vigilance over a pharmacological intervention,^26^ while one of those experiencing MCI found an improvement in objectively‐assessed inhibition, a domain of executive function,^22^ and the other single‐armed trial of those with MCI found no change in cognitive performance.^23^ The discrepancy in outcomes for older adults by cognitive status suggests that either those with a degree of cognitive impairment stand to benefit cognitively from CBT‐I or perhaps simply that the cognitive assessments are not sensitive enough to detect improvements within those of normal cognition. It is also possible that a longer period with improved sleep is needed to change objectively assessed cognitive performance.

Another important distinction among trials assessing the impact of CBT‐I on cognitive performance is the variability in assessments and cognitive domains measured. It is quite possible that the benefits of CBT‐I on cognition are domain‐specific. Two of the trials with positive results found benefits to attention,^25^, ^51^ which was integrated into the RBANS total score but not investigated as a domain of its own in this study. Additionally, while speed of information processing was the primary outcome of this trial, literature has emerged since the launch of our study that suggests performance speed may decrease with successful insomnia intervention due to correction of hyperarousal that is often seen in insomnia.^53^ In fact, faster reaction in simple vigilance tasks in those with insomnia compared to those without have been observed.^54^ Additionally, while we only investigated objective measures of cognitive performance, self‐reported indices may provide additional insights. A meta‐analysis found non‐pharmacological therapies directed at insomnia had beneficial impacts on cognitive performance; however, while effects were seen in self‐reported memory, attention, and activities of daily living, only psychomotor performance demonstrated a significant improvement among objective measures.^55^ Future studies would benefit from both objective and self‐report measures of cognitive function.

We detected a small but significant inverse correlation between change in REM time and change in raw interference, digits backward raw score, and executive function, which indicates reduced REM time is related to poorer performance in the digits backward and executive function tasks, but improved ability to inhibit an automated response. Thus, in those that did have a lengthening of REM sleep, the ability to manipulate information held in working memory (i.e., Digits Backward Raw Score) and executive function slightly declined, perhaps owing to a decrease in hyperarousal. Similarly, we detected a positive relationship between change in N2 sleep and change in HRT standard deviation, suggesting that as N2 time gets longer, response times become more variable due to either inconsistent processing speed or attention. Those with insomnia display shorter REM time^11^ and increases in N2 and REM sleep time have previously been reported with CBT‐I,^21^ although this was not detected in our cohort. Importantly, however, these findings should be interpreted with caution as they had rather small effects and were exploratory findings that were not corrected for multiple comparisons. Nevertheless, while cognitive performance per se did not improve with CBT‐I, the possibility remains that the intervention could have brough benefits to brain function (i.e., reductions in hyperarousal) that may be pertinent to the aging brain.

Contrary to our hypothesis, we did not see a benefit to Aβ accumulation nor a relationship between change in SWS and Aβ. To our knowledge, we are the first to investigate the impact of CBT‐I on Aβ, though the theoretical framework is intact as sleep, particularly SWS, contributes to the removal of Aβ.^10^ It is possible, therefore, that the lack of impact on Aβ accumulation is related to the lack of impact of the intervention on SWS. Additionally, assessing sleep microarchitecture may have shed better light on the relationship between change in SWS and Aβ as more recent evidence has suggested low frequency slow wave activity (< 1 Hz) is most relevant to Aβ burden.^56^, ^57^ Future studies may benefit from a more specific evaluation of the relationship between SWS microarchitecture and Aβ deposition following insomnia intervention. While a direct impact of CBT‐I on Aβ accumulation was not found, there remains a possibility that the improvement in insomnia symptoms following the intervention may still benefit the trajectory toward dementia in a way that is Aβ‐dependent. The average Aβ burden in this cohort was 50.1 ± 37.3 CL, suggesting amyloid pathology in most participants,^50^ although they are cognitively normal. This is important since those with insomnia and elevated Aβ experienced more rapid cognitive decline than those with just one trait.^6^ Therefore, it may be that 1 year is insufficient to see a change in Aβ deposition and longer term follow up is needed to determine if the reduction in insomnia symptoms following CBT‐I conferred any benefit to dementia progression, particularly among those with elevated Aβ.

The significant improvements in ISI that persisted up to 1 year in the CBT‐I group are consistent with the moderate effects reported in a meta‐analysis.^58^ Similarly, the lack of change in PSG‐derived sleep characteristics is consistent with systematically reviewed literature.^59^ While these two points seem in contrast, it reiterates that self‐report and objective sleep measures are distinct constructs that need not be conflated, nor should the importance of self‐report improvements in the absence of objective improvements be dismissed. In fact, diagnosis for insomnia is reliant upon self‐report, not objective measures, of sleep and daytime disturbances. Self‐reported sleep disturbances have been associated with increased risk in dementia, including AD,^2^ and insomnia is specifically associated with an increased risk of AD.^2^ This suggests that CBT‐I had a significant impact on a risk factor for dementia. Furthermore, the increase in SSE may help support continued improvement in insomnia over time. While the targeted outcomes of cognitive performance and Aβ deposition had not improved by 1 year following the intervention, long term follow up is warranted to understand how this beneficial change impacts overall dementia progression.

This study was strengthened by its gold standard RCT design. While the nature of a behavioral intervention prevents it from being fully blinded, measures were taken to blind researchers during data collection to minimize bias. Advanced technologies were used in assessing outcomes including PSG for sleep and PET/MRI for Aβ. Many confounding variables were assessed including APOE genotype and anxiety/depressive symptoms. The study is limited in that it is a pilot study with the primary focus of generating effect sizes to inform powering future studies, thus larger studies are warranted. Additionally, PSG was only performed one night, thus those outcomes may be influenced by the first night effect. The sample was predominantly female, White, non‐Hispanic/Latino, and educated, thus limiting the generalizability of our findings. Since the subsample of Aβ analysis was voluntary, there may be selection bias in that sample; however, that sample was also well‐balanced for relevant factors, including randomization. The frequency of elevated amyloid > 60% possibly reflects the inclusion criteria of the study, or a selection bias of people interested in cognitive interventions. Additionally, future investigation in those of greater symptomology (e.g., ISI ≥ 15 or some degree of cognitive impairment) may yield differing results. While our study presents data up to 1‐year post intervention, future studies would benefit from longer follow‐up to evaluate ongoing impact of CBT‐I on cognitive function, Aβ burden, and, ultimately, dementia risk.

### 4.1 Conclusions

CBT‐I did not lead to improvements in cognitive performance or Aβ deposition in cognitively normal older adults by 1 year following the intervention. However, those that received the CBT‐I experienced improvements in insomnia symptoms. Given the association between insomnia and AD, future investigation is warranted to understand the long‐term impact CBT‐I on the progression of AD.

## CONFLICT OF INTEREST STATEMENT

C.S. is owner and CEO of Sleep Health Education, L.L.C.; L.H., E.N., A.G., R.L., J.R., M.P., J.D., J.B., E.V., M.D., J.M., and J.M.B. have no conflicts of interest to disclose. Author disclosures are available in the Supporting Information.

## CONSENT STATEMENT

All participants underwent informed consent.

## Supporting information




**Supporting Information**: alz71591‐sup‐0001‐Appendix‐1.docx

**Supporting Information**: alz71591‐sup‐0002‐ICMJE.pdf

## Data Availability

Data used in this manuscript are available upon reasonable request to the corresponding author.
