# Supplementary material for: The impact of cognitive behavioral therapy for insomnia on cognitive performance and amyloid beta in older adults: A randomized controlled trial
Source: Alzheimers Dement. 2026 Jun 15;22(6):e71591. doi: 10.1002/alz.71591 (PMC13269014; doi:10.1002/alz.71591)
Supplement: Supplementary file 1 — Supporting Information: alz71591‐sup‐0001‐Appendix‐1.docx [file ALZ-22-e71591-s001.docx]

| **Supplemental Table 1. Change in Sleep Characteristics in CBT-I vs Active Control** | | | | | | | | | | | | |
| --- | --- | --- | --- | --- | --- | --- | --- | --- | --- | --- | --- | --- |
|  | **Active Control** | | | **CBT-I** | | | **Between-group difference in change at 6 weeks** | | **Between-group difference in change by 1 year** | | |  |
| **Outcome** | **Baseline**  **Mean±SD**  **(n=100)** | **6 Weeks**  **Mean±SD**  **(n=86)** | **1 Year Mean ±SD**  **(n=81)** | **Baseline**  **Mean±SD**  **(n=100)** | **6 Weeks Mean±SD**  **(n=89)** | **1 Year**  **Mean±SD**  **(n=86)** | **P-value** | **Test** | **P-value** | **Test** |  |  |
| Sleep Onset (min) | 26.5±28.4 | 21.3±25.9 | 19.2±19.2 | 20.0±20.3 | 27.2±30.3 | 20.3±22.1 | 0.0123 | Rank sum | 0.5397 | Rank sum |  |  |
| Time in Bed (min) | 442.8±41.9 | 437.6±29.3 | 440.9±30.5 | 436.6±37.8 | 435.2±31.0 | 439.3±32.5 | 0.9002 | Rank sum | 0.8611 | Rank sum |  |  |
| Total Sleep Time (min) | 329.3±68.6 | 337.4±57.2 | 350.6±52.9 | 319.0±74.3 | 319.5±67.8 | 341.3±32.5 | 0.3131 | T-test | 0.3157 | Rank sum |  |  |
| REM Time (min) | 60.7±33.3 | 60.3±32.0 | 64.6±27.0 | 50.6±31.6 | 57.0±28.0 | 58.2±28.3 | 0.043 | Rank sum | 0.9529 | Rank sum |  |  |
| REM (%) | 17.8±8.2 | 17.5±8.3 | 18.1±6.6 | 15.0±7.6 | 17.4±7.4 | 16.8±7.4 | 0.0208 | T-test | 0.5411 | Rank sum |  |  |
| Sleep Efficiency (%) | 74.4±14.4 | 77.1±11.8 | 79.4±10.2 | 72.9±15.0 | 73.5±14.8 | 77.8±50.2 | 0.2887 | Rank sum | 0.3681 | Rank sum |  |  |
| WASO (min) | 86.8±53.8 | 78.3±48.7 | 70.7±41.0 | 97.1±59.4 | 88.2±56.8 | 77.4±50.2 | 0.9476 | Rank sum | 0.7583 | Rank sum |  |  |
| N1 (min) | 18.6±15.2 | 24.8±20.0 | 24.0±16.8 | 25.2±22.9 | 23.0±19.0 | 22.8±19.3 | 0.0271 | Rank sum | 0.1002 | Rank sum |  |  |
| N1 (%) | 6.0±5.1 | 7.6±6.2 | 7.2±6.0 | 8.5±8.3 | 7.6±6.7 | 6.8±5.5 | 0.0492 | Rank sum | 0.1139 | Rank sum |  |  |
| N2 (min) | 198.4±57.5 | 209.0±48.0 | 219.2±49.4 | 193.0±62.6 | 192.6±57.5 | 216.0±56.8 | 0.1956 | T-test | 0.7666 | T-test |  |  |
| N2 (%) | 60.5±13.2 | 64.7±24.9 | 62.8±11.8 | 60.8±14.0 | 60.5±13.3 | 63.2±12.8 | 0.4493 | Rank sum | 0.9213 | T-test |  |  |
| N3/SWS Time (min) | 51.5±37.1 | 43.2±32.1 | 42.9±36.8 | 50.2±35.9 | 46.9±37.6 | 44.3±37.6 | 0.8147 | Rank sum | 0.5135 | Rank sum |  |  |
| N3 (%) | 15.8±11.0 | 12.6±8.7 | 11.9±10.0 | 15.7±10.4 | 14.6±10.9 | 13.2±11.4 | 0.614 | Rank sum | 0.9127 | Rank sum |  |  |

Note: REM- Rapid Eye Movement; NREM-Non-Rapid Eye Movement; WASO- Wake after sleep onset; N1- sleep stage 1; N2- sleep stage 2; N3/SWS- sleep stage 3/slow wave sleep; AHI- apnea-hypopnea index; PLM- periodic limb movement; PLMS- periodic limb movement of sleep
